# Immunological Consequences of Antihelminthic Treatment in Preschool Children Exposed to Urogenital Schistosome Infection

**DOI:** 10.1155/2013/283619

**Published:** 2013-06-05

**Authors:** Nadine Rujeni, Norman Nausch, Nicholas Midzi, Graeme J. Cowan, Richard Burchmore, David R. Cavanagh, David W. Taylor, Takafira Mduluza, Francisca Mutapi

**Affiliations:** ^1^Institute of Immunology and Infection Research, School of Biological Sciences, Ashworth Laboratories, University of Edinburgh, King's Buildings, West Mains Road, Edinburgh EH9 3JT, UK; ^2^National Institute of Health Research, Box CY 570, Causeway, Harare, Zimbabwe; ^3^Research Council of Zimbabwe, Block A, Delken Complex, Mount Pleasant Business Park, Harare, Zimbabwe; ^4^Institute of Infection, Immunity and Inflammation, University of Glasgow, Sir Graeme Davies Building, 120 University Place, Glasgow G12 8TA, UK; ^5^Division of Pathway Medicine, School of Biomedical Sciences and Edinburgh Infectious Diseases, Ashworth Laboratories, University of Edinburgh, King's Buildings, West Mains Road, Edinburgh EH9 3JT, UK; ^6^Department of Biochemistry, University of Zimbabwe, P.O. Box 167, Mount Pleasant, Harare, Zimbabwe

## Abstract

Urogenital schistosomiasis, due to *Schistosoma haematobium, *is endemic in sub-Saharan Africa. Control is by targeted treatment with praziquantel but preschool age children are excluded from control programs. Immunological studies on the effect of treatment at this young age are scarce. In light of studies in older individuals showing that praziquantel alters antischistosome immune responses and responses to bystander antigens, this study aims to investigate how these responses would be affected by treatment at this young age. Antibody responses directed against schistosome antigens, *Plasmodium falciparum *crude and recombinant antigens, and the allergen house dust mite were measured in children aged 3 to 5 years before and 6 weeks after treatment. The change in serological recognition of schistosome proteins was also investigated. Treatment augmented antischistosome IgM and IgE responses. The increase in IgE responses directed against adult worm antigens was accompanied by enhanced antigen recognition by sera from the children. Antibody responses directed against *Plasmodium *antigens were not significantly affected by praziquantel treatment nor were levels of allergen specific responses. Overall, praziquantel treatment enhanced, quantitatively and qualitatively, the antiworm responses associated with protective immunity but did not alter *Plasmodium*-specific responses or allergen-specific responses which mediate pathology in allergic disease.

## 1. Introduction

Schistosomiasis is a major human helminth infection endemic in developing countries where children harbor the greatest number of parasites. Urogenital schistosomiasis, caused by infection with *S. haematobium, *is the most prevalent in sub-Saharan Africa and affected children suffer stunted growth, impaired memory and cognitive development, anemia, haematuria, and reduced physical fitness. Current control programs target school children, typically aged 6 years old and above and younger pre-school age children (i.e., 5 years and below) are not treated. This is partly because they have been regarded as being at very low risk of infection and infection-associated morbidity and partly because, until recently, there has been lack of safety data on praziquantel in this age group [1]. Yet, the reality is that exclusion of these children from control programs leaves them at high risk of developing chronic pathology as has been shown in high endemic foci [2]. 

This health inequality is now being addressed with the realization that preschool age children do experience significant levels of infection [3–7]. The WHO has recently formally recognized that these children should be considered for treatment [8] and studies on the safety and efficacy of praziquantel, as well as methods of diagnosis and drug delivery in this age group [6, 9–11], have been carried out. The next stage is to assess the consequences of praziquantel treatment beyond the reduction of infection, particularly the effects on schistosome-specific responses as well as responses to bystander antigens. 

Indeed, praziquantel treatment of school children and adults has been shown to enhance immune responses associated with resistance to reinfection with schistosomes in endemic areas [12, 13] but immunological studies in pre-school children are scarce. Moreover, antihelminthic treatment has been associated with an increase in atopy [14, 15], as a result of alterations in the immunological balance established by the parasites. These studies were conducted in schoolchildren and the effect of antihelminthic treatment on allergic sensitization in younger children remains to be determined. 

Schistosome infections have also been reported to both exacerbate and ameliorate responses associated with resistance to *Plasmodium falciparum,* the causative agent of malaria [16, 17]. Nonetheless, previous studies in schoolchildren aged 6–18 years old showed that antihelminthic treatment of *S. haematobium* infection did not alter immune responses to the malaria vaccine candidates merozoite surface protein (MSP) 1 and 2 [18]. It remains to be determined if this is the case in younger children. 

Thus, the first aim of this study was to determine the short-term antihelminthic effects on immunological markers of exposure to schistosome (IgM directed against schistosome cercariae, egg, and adult worms [19]) and malaria (IgM directed against *Plasmodium* total schizont [20]) infections as well as the effects on antibody responses associated with resistance to infection/reinfection (IgE and IgG4 directed against schistosome cercariae, egg, and adult worms, IgG directed against *Plasmodium* vaccine candidates MSP-1 and MSP-2 [16, 21–23]) in children aged 3–5 years in Zimbabwe. We also determined the effects of antihelminthic treatment on allergic sensitization to the house dust mite *D. pteronyssinus*, the most prevalent allergen in Zimbabwe [24].

 The second aim of this study was to identify antigens serologically recognized on the adult worm by these children (individually) both before and 6 weeks after treatment, to assess whether antischistosome antibody changes following treatment were due to qualitative changes in antigens recognised by the host immune system as has been shown in adults [25].

## 2. Materials and Methods

### 2.1. Study Population

The study population was recruited from Magaya, Chitate, and Chipinda villages in the Mashonaland East Province of Zimbabwe where *S. haematobium* is endemic. Populations from the 3 villages and selection criteria have been described elsewhere [9, 26, 27]. Forty-one (41) children were included in this study; 31 had received praziquantel treatment while 10 children, whose parents declined treatment for religious reasons, formed the untreated control group. All the treated children who were egg positive at baseline (*n* = 4) had cleared infection at the six weeks follow up. There was no significant difference between treated/untreated children in terms of village of residence (*Z* = −1.037, *P* = 0.463) and their parents were all subsistent farmers. All the children were also part of a larger study investigating the safety of praziquantel and they reported no adverse effects 24 hours following treatment [9]. Three children provided enough blood samples to enable proteomic analyses on schistosome antigens serologically recognised before versus after treatment. One of them was in the untreated group and served as a negative control. All the 3 children were excreting schistosome eggs in urine at baseline.

### 2.2. Ethical Statement

Permission to conduct the study in the region was obtained from the Provincial Medical Director. Institutional and ethical approval was received from the University of Zimbabwe and the Medical Research Council of Zimbabwe respectively. In addition, the study received ethical approval from the World Health Organization's Research Ethics Review Committee. At the beginning of the study, parents and guardians of participating children had the aims and procedures of the project explained fully in the local language, Shona, and written consent was obtained from participants' parents/guardians before enrolment into the study. After collection of all samples, all compliant participants were offered anti-helminthic treatment with the recommended dose of praziquantel (40 mg/kg of body weight).

### 2.3. Antigens

Lyophilized soluble* S. haematobium *adult (SWAP), cercariea (CAP), and egg (SEA) antigens were obtained from the Theodor Bilharz Institute (Giza, Egypt) and reconstituted in ultra pure water as recommended by the manufacturer. *D. pteronyssinus* Derp1 allergen was obtained from Indoor Biotechnologies. Crude *P. falciparum* schizont antigens were of the Wellcome strain of the parasite. Recombinant antigens (from *Plasmodium* merozoite surface protein, MSP) were MSP-1_19_ antigen, the 19 kDa C-terminal domain of MSP1, and MSP-2 which has 2 serotypes: CH150 that belongs to serotype A (3D7-like), and Dd2 that belongs to serotype B (FC27-like) [28]. 

### 2.4. Antibody Assays

A standard indirect enzyme-linked immunosorbent assay (ELISA) was optimized as described previously [26, 27] and used to quantify levels of antibodies directed against SWAP, SEA, and CAP (IgM, IgE, and IgG4); *P. falciparum* antigens (IgM and IgG), and Derp 1 allergen (IgE and IgG4) both before and 6 weeks after treatment. 

For schistosome-specific antibodies, microtitre plates were coated overnight at 4°C with 100 *μ*L/well of antigen at 5 *μ*g/mL (10 *μ*g/mL for SEA) in carbonate bicarbonate buffer (pH = 9.6) and then washed once with PBS/0.03% Tween20 (which was used for all subsequent washes). Plates were blocked with 5% skimmed milk in PBS/0.03% Tween20. 100 *μ*L of the serum samples was then added diluted at 1 : 20 for IgE (anti-SEA, SWAP and CAP); 1 : 100 for IgM anti-SWAP and IgG4 anti-CAP and SWAP; 1 : 400 for IgM anti-CAP and SEA and IgG4 anti-SEA. Plates were incubated for 2 hours at 37°C. After washing 3 times, 100 *μ*L of antihuman horse-radish peroxide conjugated IgE (Sigma), IgM (Dako) or IgG4 (The Binding Site) was added diluted (with blocking buffer) at 1 : 1000 except for IgM anti-SWAP (1 : 2000), IgG4 anti-CAP and SWAP (1 : 500), and IgE anti-SWAP (1 : 250). After 1 hour incubation at 37°C followed by 6 washes, 100 *μ*L of the substrate (ABTS) was added. The reaction was stopped, using 25% HCL, after 15 min incubation at room temperature for IgM and IgG4 and 30 min incubation at 37°C for IgE. Absorbance readings of all wells were performed at 405 nm. 

For *Plasmodium-*specific responses, the same protocol was followed but 50 *μ*L/well of 1 *μ*g/mL (crude schizont) or 50 ng/mL (recombinant antigens) was used to coat plates. All samples were diluted 1 : 100 for all assays and secondary antibodies (Sigma) diluted 1 : 1000 (IgM and IgG) for crude antigens and 1 : 2000 (IgG) for recombinant antigens. The colorimetric reaction time was 15 minutes at room temperature for all assays. 

For Derp1-specific responses, plates were coated with 50 *μ*L/well of 5 *μ*g/mL and samples diluted 1 : 10. Detection antibodies (IgE from Sigma and IgG4 from The Binding Site) were diluted 1 : 1000 and the reaction time for the substrate was 30 minutes at 37°C. 

All laboratory assays for the pre- and posttreatment time points were run at the same time and by the same individual. All samples were assayed in duplicate and 3 sera from European children (negative controls) who have never been exposed to schistosome or malaria infections were included on each plate and were used to set up cutoff points for IgM status (ODs of 0.2, 0.24, and 0.55, resp., for SWAP, CAP, and SEA). A blank control containing no sera was included in duplicate on each plate and the background absorbance of reagents in the absence of serum was subtracted from all readings. A pool of responders (positive controls) was run on each plate and readings were comparable across plates; that is, the % CVs were less than 20% for all comparisons and readings were below the critical values as determined by Reed et al. [29]; therefore no correction factor was used. Antibody levels were expressed as optical densities.

### 2.5. Gel Electrophoresis

A 1-dimensional gel separation was performed. One hundred *μ*g of antigen (SWAP), diluted 1 : 1 in lithium dodecyl sulphate, was run on a 10% gel using sodium dodecyl sulphate (SDS) buffer. The gel (7 cm) was subsequently divided longitudinally into 2 parts, the first to be used for Western blotting and the second for protein identification. The same gel for Western blot and protein identification was used to exclude variations due to experimental settings. The proteins on the piece of gel used for identification were stained with silver nitrate while the rest were transferred onto a 0.45 *μ*m nitrocellulose membrane.

### 2.6. Western Blotting

Proteins were transferred onto a nitrocellulose membrane using a wet system, in transfer buffer made of 20% methanol, 25 mM Tris, and 192 mM Glycine. The membrane was then blocked with 2% BSA in TBS/0.05% Tween20 for 3 hours, washed 2 times in TBS/0.05% Tween/0.2% triton x-100, and cut into strips. On each strip, 1.5 mL of individual serum diluted 1 : 50 in blocking solution was added and all strips were incubated overnight at 4°C. After 3 washes, 1.5 mL of HRP-conjugated IgE antibody (Sigma) was added and the strips incubated for 2 hours at room temperature. After 4 washes, the strips were incubated in ECL Plus reagent system for 5 minutes, before being exposed on hyperfilm. The film was developed and bands were matched to those on the silver-stained piece of gel. 

### 2.7. Mass Spectrometry

Bands on the silver stained piece of gel that matched those on the Western blots were excised, reduced with Farmer's reagent, and subjected to in-gel trypsin digestion using standard protocols [30]. The resulting tryptic peptides were solubilized in 0.5% formic acid and fractionated on a nanoflow uHPLC system (Thermo RSLCnano). Peptide separation was performed on a Pepmap C18 reversed phase column (LC Packings), using a 5–85% v/v acetonitrile gradient (in 0.5% v/v formic acid) run over 45 min at a flow rate of 0.2 uL/min. Eluate was analysed by online electrospray ionisation (ESI) mass spectrometry using an Amazon ion trap MS/MS (Bruker Daltonics) operating a continuous duty cycle of survey MS scan followed by up to five MS/MS analyses of the most abundant peptides, choosing the most intense multiply charged ions with dynamic exclusion for 120 s.

MS data was processed using Data Analysis software (Bruker) and the automated Matrix Science Mascot Daemon server (v2.1.06). Protein identifications were assigned using the Mascot search engine to interrogate Schistosome protein sequences in the NCBInr database, allowing a mass tolerance of 0.3 Da for both MS and MS/MS analyses. 

### 2.8. Statistical Analysis

Since the data did not fulfill the assumptions for parametric tests and data transformations could not overcome this, nonparametric tests were used for all statistical analyses. The nonparametric Wilcoxon signed-rank tests for paired samples were performed to compare pre- and posttreatment antibody raw data (optical densities), ratios (IgE/IgG4), and infection intensities. For comparisons between groups of different individuals (i.e., infected versus uninfected, IgM negative versus IgM positive, etc.), Mann-Whitney *U* tests were performed. The cutoffs for IgM anti-schistosome or antischizont antigens were calculated as the mean absorbance plus 2 standard deviations of readings from European negative controls. All statistical analyses were conducted in PASW statistics (formerly SPSS) 17 and *P* values of <0.05 were considered significant. A sequential Bonferroni correction was used to identify results that were significant in the context of the multiple statistical comparisons [31].

## 3. Results

### 3.1. Infection and Antibody Levels before Antihelminthic Treatment

At baseline, 38 children provided urine samples and 13.2% (CI_95_ 4.4–28) were schistosome egg positive (mean egg count of 10 eggs/10 mL). Of the 41 children included in this study, 17.5% (CI_95_ 7.3–32.8) showed immunological evidence of recent exposure to egg antigens (positive anti-SEA IgM), 39% (CI_95_ 24.2–55.5) to cercariae antigens (positive anti-CAP IgM), and 22% (CI_95_ 10.6–37.6) to adult schistosome antigens (positive anti-SWAP IgM).

None of the children were *P. falciparum* positive as determined by thin and thick smears or by the Paracheck serological test but 21% (CI_95_ 7.1–42.1) showed immunological evidence of recent exposure to parasite antigens (positive anti-schizont IgM). No significant difference in *Plasmodium*-specific antibody levels was observed between schistosome egg positive versus egg negative children (*Z* = −0.132, *P* = 0.935; *Z* = −0.742, *P* = 0.505; *Z* = −0.579, *P* = 0.62; *Z* = 1.354, *P* = 0.202; *Z* = −1.180, *P* = 0.271, resp., for MSP119, DD2, CH150, Schizont-IgM, and Schizont IgG). Similarly, there was no significant difference in levels of Derp1-specific responses between schistosome egg positive versus egg negative (*Z* = −0.173, *P* = 0.4315; *Z* = −1.152, *P* = 0.1245; *Z* = −1.36, *P* = 0.087, resp., for IgE, IgG4, and IgE/IgG4 ratio). 

### 3.2. Effects of Praziquantel Treatment on Antibody Levels

At baseline, there was no significant difference in mean antibody levels (all isotypes) between children who were subsequently treated and those who were not. Furthermore, infection levels were comparable between the two groups (*Z* = −0.146, *P* = 0.884 for mean infection intensity and *χ* ^2^ = 0.043, *P* = 0.662 for prevalence).

Six weeks after treatment, all of the treated children were schistosome egg negative (all the egg positive children had cleared infection), resulting in a significant fall in the mean egg count (*Z* = −1.626, *P* = 0.034). On the other hand, infection intensity increased for the one child who was egg positive at baseline in the untreated group (73 to 166 eggs per 10 mL). There were no newly infected children in the untreated group. There were significant changes in levels of the majority of antibodies in the treated children between the two time points as shown in Table 2 and detailed below. Overall, the percentage of children with positive CAP- and SWAP-IgM (marker of exposure) increased after treatment (39% to 63.4% and 22% to 43.9%, resp.).

#### 3.2.1. Schistosome-Specific Antibody Levels

**Figure 1:**
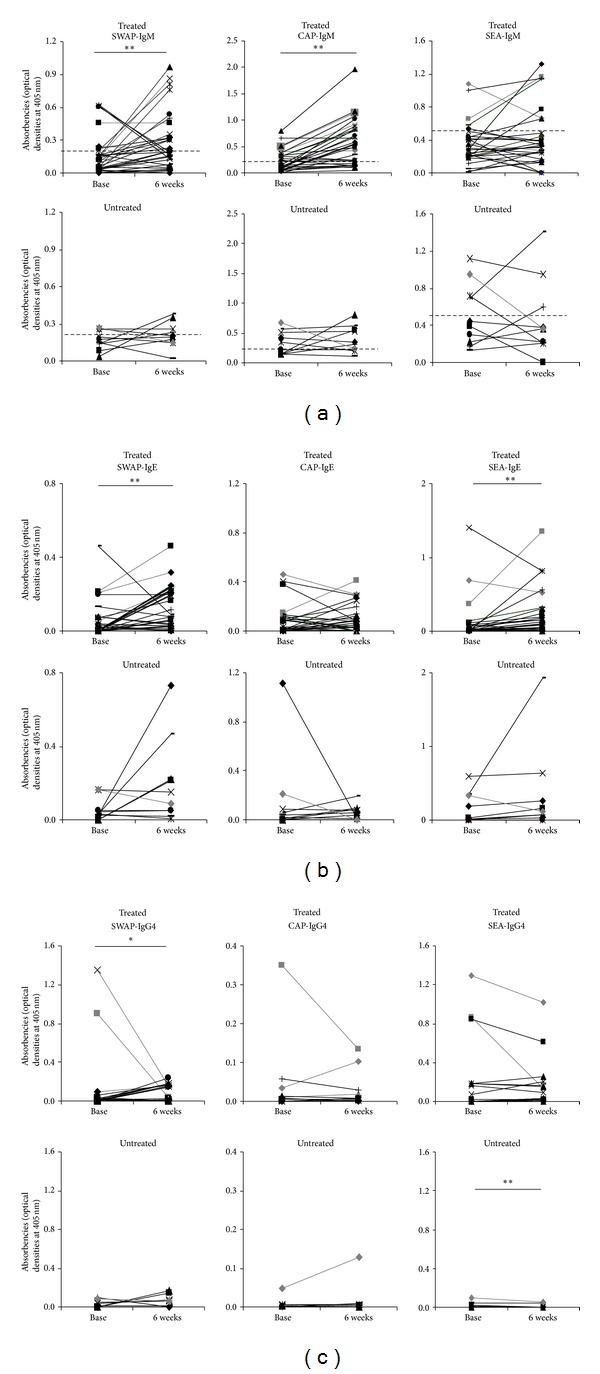
Change in anti-schistosome antibody titers (optical densities). Serum IgM (a); IgE (b) and IgG4 (c) against adult worm (SWAP), cercariae (CAP), and egg (SEA) antigens are plotted as lines connecting pre- (baseline) and post- (6 weeks) treatment levels for treated and untreated children. Participants who were egg positive at baseline are highlighted in grey thick lines. Stars represent significant difference of mean absorbencies at **P < 0.01 or *P < 0.05. The cut-off points for IgM are highlighted with dashed horizontal lines (a).

Adult schistosome worm and cercariae-specific IgM titers, but not egg-specific IgM, rose significantly in treated children (Figure 1(a)). Similarly, adult worm and egg-specific IgE titers also increased significantly following treatment (Figure 1(b)). The figures (Figures 1(a) and 1(b)) further illustrate that there was no clear distinction between children who were egg positive and negative at baseline. 

Overall, adult worm specific IgG4 titers (Figure 1(c)) decreased following treatment, but the decline only occurred in children with mild infection at baseline (0.3 to 10.7 mean eggs/10 mL) and not in egg negative children. The decline was however not statistically significant after Bonferroni correction (Table 2). 

In contrast, levels of all anti-schistosome antibodies (except egg-specific IgG4 which declined) did not change significantly in the 6 weeks period for untreated children (Figures 1(a)–1(c) and Table 1). No significant changes occurred in the ratio IgE/IgG4 against all the 3 parasite life cycle stages (*Z* = −1.118, *P* = 0.453; *Z* = −1.139, *P* = 0.128; and *Z* = −0.02, *P* = 0.492, resp., for adult worm, cercariae and egg antigens) in either group (treated or untreated). 

#### 3.2.2. Derp1-Specific Antibody Levels

**Figure 2:**
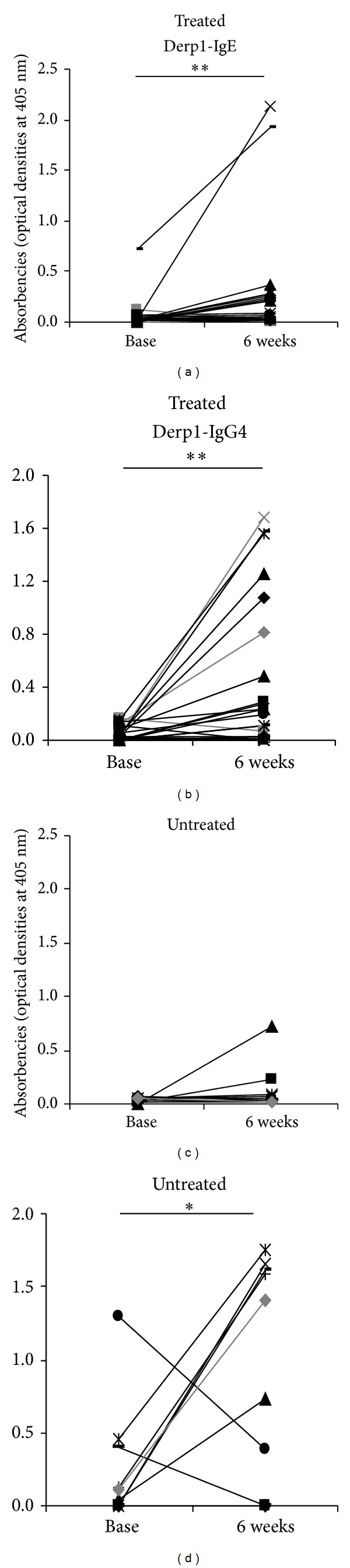
Change in anti-Derp1 antibody levels. Serum IgE and IgG4 (optical densities) against Derp1 allergen are plotted as lines connecting pre- (baseline) and post- (6 weeks) treatment levels for treated and untreated children. Participants who were egg positive at baseline are highlighted in grey thick lines. Stars represent significant difference of mean absorbencies at *P < 0.05 or **P < 0.01.

A significant increase in IgE and IgG4 against Derp1 was observed in treated children (Figure 2). However, an increase in IgG4 was also observed in untreated controls. No significant change was observed in the ratio IgE/IgG4 in both groups (*Z* = −0.431, *P* = 0.333 and *Z* = −0.357, *P* = 0.361, resp., for treated and untreated groups). 

#### 3.2.3. Plasmodium-Specific Antibody Levels

**Figure 3:**
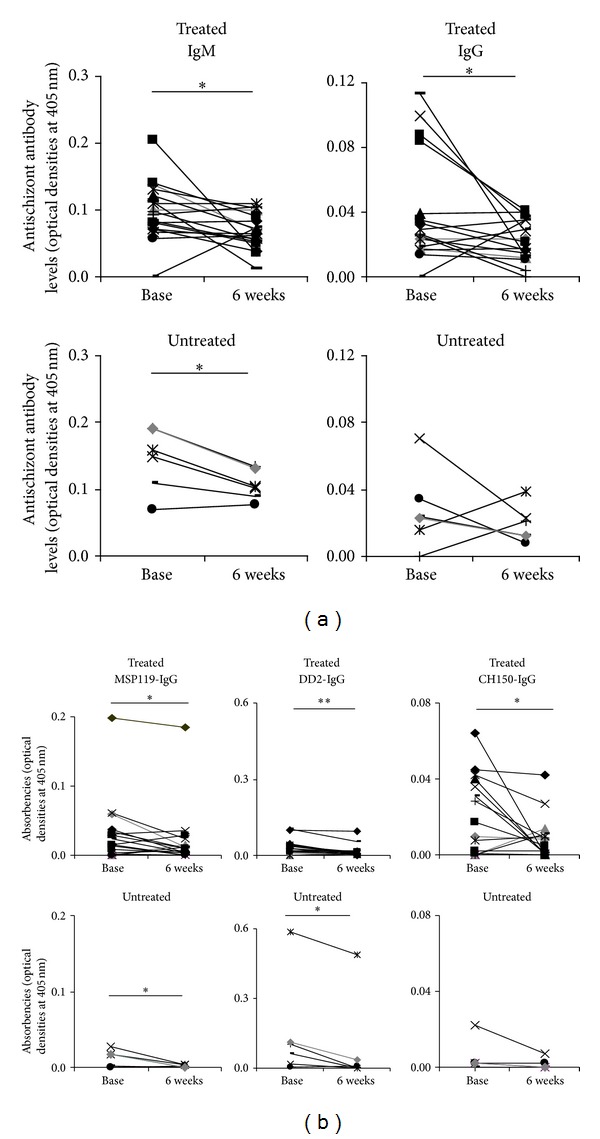
Change in anti-*Plasmodium-falciparum* antibody levels. (a) *P. falciparum* schizont-specific IgM and IgG (optical densities) and (b) antibody levels (IgG) against the merozoite surface proteins MSP-1 (MSP1_19_) and MSP-2 (Dd2 and CH150) are shown as lines connecting pre- (baseline) and post- (6 weeks) treatment levels for treated and untreated children. Participants who were egg positive at baseline are highlighted in grey thick lines. Stars represent significant difference of mean absorbencies at *P < 0.05 or **P < 0.01.

A decrease occurred in titres of IgM and IgG antibodies against the total schizont antigen following praziquantel (PZQ) treatment (Figure 3(a)). Similarly there was a significant decline in IgG levels against all the malaria vaccine candidates MSP-1 (MSP1_19_) and MSP-2 (Dd2 and CH150) after treatment (Figure 3(b)). Untreated children also exhibited a decrease in IgG against Dd2 and MSP1_19_, and IgM against the total schizont over the same time frame. 

### 3.3. Antigens Recognized before versus after Treatment

**Figure 4:**
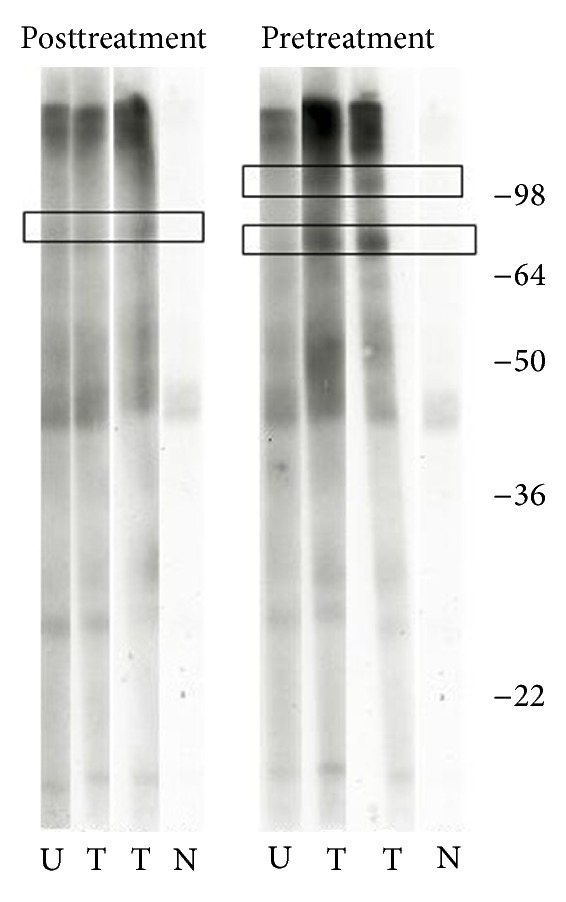
Immunoblots of serum samples. Comparison of pre- (left) and post- (right) treatment immunoblots detected by their reactivity with IgE antibodies. Identified bands are shown in boxes and molecular weights (in kDa) are indicated at the right end. Strips were exposed on hyperfilm for seconds (touch exposure). U: untreated, T: treated, and N: assay control (no sample added).

Gel bands were detected by their reactivity with IgE antibodies in serum samples. The before treatment and after treatment Western blot assays were conducted on the same membrane to exclude any variation that might arise from the use of different antigen preparations. Only 1 band (~80 kDa) was recognized by all serum samples collected before treatment (Figure 4). Six weeks following treatment, sera from treated children recognized an additional band (~100 kDa) which was absent on the blot from the untreated child. Furthermore, the image master analysis showed that the 80 kDa band was enhanced after treatment (the bands were quantified by their pixel intensities). The potential identities of the proteins recognized, and the corresponding hit scores, are listed in Table 3. 

## 4. Discussion 

With increasing calls for the inclusion of pre-school children and infants in mass chemotherapy programmes for schistosome control [1, 4, 6, 9, 11], and studies in school children and adults suggesting that antihelminthic treatment not only affects immune responses directed against schistosomes but may also affect immune responses directed against unrelated antigens [13, 14, 32], the current study aimed to investigate immunological consequences of praziquantel treatment in young pre-school age children. 

The study shows significant changes in the levels of anti-schistosome adult worm IgM and IgE antibody titres 6 weeks following praziquantel treatment in children aged 3–5 years old living in an *S. haematobium* endemic area. Anti-schistosome egg IgE and anticercaria IgM also increased significantly following treatment. There was a decline in anti-schistosome worm IgG4 levels following treatment, essentially driven by children presenting with *S. haematobium* eggs in urine at baseline. However, this decline was not statistically significant following Bonferroni correction, possibly due to the small sample size. 

A relatively small number of participants in this study were egg positive at baseline. The change in anti-schistosome antibody levels in children who were egg negative at baseline suggests that these children may have had prepatent infection, single sex infection, and/or low levels of infection undetected by the egg count technique [33]. Indeed, recent studies in pre-school children using a rapid diagnostic test based on cercariae antigens indicate that infection levels are about 3 times higher than prevalence obtained by the egg count [34]. Consistent with these findings, the current study shows a prevalence of 39% based on CAP-IgM, which is 3 times higher than the 13% prevalence obtained based on egg count. The possibility of prepatent and/or single sex infections was further supported by the increase in the percentage of children with positive CAP- and SWAP-IgM after treatment.

The increase in anti-worm and anti-cercariae IgM responses is consistent with reported changes in adult worm (and cross-reactive) antigens exposed to the immune system following the killing of adult worms by praziquantel [35]. Antiegg responses have been proposed as a diagnostic tool for schistosome infections in young children where egg counts are less reliable due to low levels of patent infection or to single sex infections [11]. In this study, antiegg IgM responses did not change following treatment, suggesting that while antiegg responses may be good markers of the presence of a patent schistosome infection before treatment, they may not be a reliable tool for use in evaluating the efficacy of treatment.

This study showed that, consistent with findings in older individuals [32], praziquantel treatment boosts the anti-worm IgE immune responses associated with protection, suggesting that this young age group may also benefit immunologically from anti-schistosome chemotherapy, which reduces reinfection in older children and adults by stimulating IgE responses [13, 32]. 

The increase in IgE titres was accompanied by an increase in the number of parasite antigens recognised, suggesting that treatment results in the enhancement of antigen recognition as already reported for older children and adults [25]. Potential mechanisms for this include the introduction of previously “hidden” antigens or enhanced affinity maturation of IgE responses. The proteins identified after treatment were essentially constituents of the parasite surface and musculature, consistent with the praziquantel disruptive action on them [36–38]. Among these was the myosin heavy chain, which has been previously recognized by pooled sera from older individuals with a longer history of infection [39]. Two distinct fragments of this protein have previously been associated with protection against reinfection in mice vaccinated with radiation-attenuated cercariae [40, 41]. *S. mansoni* heat shock protein 70 (HSP70) homologue was also identified, consistent with previous results from pooled samples of older individuals [25]. HSPs are proteins that are expressed by all eukaryotic cells to maintain cell homeostasis under stress conditions. Furthermore, because they are suspected of being inducers of multiple pathways of immunity, these proteins may be explored as potential adjuvants in vaccine development against cancer and infections (reviewed by Segal et al. [42]). 

This approach presents the limitation that all the parasite antigens described were recognised by IgE. Although parasite-specific IgE antibodies have been associated with protection [21, 32, 43], vaccine candidates eliciting IgE responses in helminth endemic areas have recently been shown to induce urticaria [44]. Although “desensitization” of the IgE-inducing vaccine is a possibility to overcome this problem, identification of antigens recognized by other isotypes [45] may be more realistic. Nevertheless, characterising parasite antigens eliciting IgE responses gives the information on what to “avoid” or “take with precaution” in vaccine design. Furthermore, given the similarities between anti-helminth and anti-allergen responses [46], comparative studies (schistosome antigens versus allergens) would provide the spectrum of antigen recognition and inform on possible cross-reactivity. This would narrow the choice of antigen vaccine candidates and minimise the risk of sensitization.

In this study, anti-*Plasmodium* responses declined in both treated and untreated children, suggesting that this change was not due to praziquantel treatment. Since the decline was both in IgM responses directed against the schizont (marker of exposure to *Plasmodium *infection) and IgG responses directed against the vaccine candidates MSP-1 and MSP-2, it is likely that the decline was reflecting normal dynamics of *Plasmodium* antibodies in the absence of infection. Levels of antibodies to several *Plasmodium* antigens vary with the seasonality of parasite transmission, often being higher during periods of high transmission than at the end of a low transmission season [47]. Furthermore, levels of anti-*Plasmodium* antibodies directed against both crude and recombinant antigens tend to be higher in individuals carrying parasites than in those without parasites [47]. The region of Zimbabwe where the present study was conducted is mesoendemic for malaria with an incidence of 0.2 to 10 cases/1000/year [48]. The absence of *Plasmodium *parasites on blood smears of participants in this study and the decline in the levels of anti-*Plasmodium* antibody responses may therefore reflect the end of a malaria transmission season in this area (at the time of sample collection).

In the present study, there was no significant difference for Derp1 specific antibodies between schistosome egg positive and negative children at baseline. Following treatment, Derp1 specific IgE and IgG4 were significantly increased, resulting in the ratio IgE/IgG4 (which is associated with allergic reactivity [49]) being not significantly changed. Hagel and colleagues indicated that a certain “threshold” is required for parasite infection to suppress allergic reactivity [50], consistent with findings from animal models [51]. The low levels of infections in the present study population would therefore explain the absence of significant changes in the ratio IgE/IgG4 against Derp 1 allergen. The increase in allergen-specific IgE responses in treated children may be due to cross-reactivity since levels of these antibodies directed against schistosome antigens also increased following treatment. However, cross-reactivity cannot explain the increase in IgG4 against Derp1 in treated and, to a lesser extent, in untreated children, since the anti-schistosome IgG4 declined. Changes in antimite IgG4 antibodies following seasonal changes in natural exposure to house dust mites have previously been reported [52]. Investigations on natural temporal variations in levels of exposure to mite were beyond the scope of this study but this finding requires further investigation. 

The current study presents the limitation that the sample size was small, which could have affected the statistical power. This was due to the limited accessibility of these young children in terms of blood collection, and also because treatment has only recently started in this age group. Nevertheless, this study is one of the first to demonstrate a boost, due to praziquantel treatment, in anti-schistosome immune responses in pre-school age children. 

## 5. Conclusion

Taken together, findings from this study suggest that praziquantel treatment of preschool children has comparable effects to those reported in studies on older children and adults, boosting antibody responses associated with putative resistance to schistosome infection/reinfection six weeks following chemotherapy. Furthermore, serological recognition of schistosome antigens was enhanced following praziquantel treatment in these young children, and recognized antigens are associated with the development of acquired protective immunity. Praziquantel treatment did not have a significant effect on *Plasmodium*-specific responses, which showed temporal fluctuation in both untreated and treated individuals. Furthermore, PZQ treatment did not significantly alter allergen-specific responses which would mediate pathology in allergic disease. Thus, the study suggests that pre-school age children may also benefit immunologically from praziquantel treatment. 

## Figures and Tables

**Table 1:** Characteristics of the study groups at baseline.

	Treated	Untreated	Difference (*Z* and *P* value or *χ* ^2^)
Mean age (years)	4.5	4.7	−1.341, 0.247
Mean infection intensity (eggs/10 mL)	10	8.1	−0.146, 0.946
(Prevalence in %)	13.2	11.1	*χ* ^2^ = 0.043,*P* = 0.835
Residence	9/14/8	7/2/1	−1.037, 0.063
Male/female	9/22	4/10	−0.640, 0.622
SWAP-IgM (OD ± SEM)	0.155 ± 0.03	0.172 ± 0.02	−1.548, 0.127
Prevalence in %	14.6	7.3	*χ* ^2^ = 0.5, *P* = 0.479
CAP-IgM (OD ± SEM)	0.225 ± 0.036	0.333 ± 0.06	−1.913, 0.057
Prevalence in %	26.8	12.2	*χ* ^2^ = 0.67, *P* = 0.4
SEA-IgM (OD ± SEM)	0.345 ± 0.04	0.513 ± 0.1	−1.312, 0.198
Prevalence in %	7.5	10	**χ** ^2^ = 4.67, **P** = 0.03
SWAP-IgE (OD ± SEM)	0.054 ± 0.02	0.054 ± 0.02	−0.972, 0.345
CAP-IgE (OD ± SEM)	0.077 ± 0.02	0.154 ± 0.1	−0.245, 0.823
SEA-IgE (OD ± SEM)	0.106 ± 0.05	0.151 ± 0.06	−0.473, 0.643
SWAP-IgG4 (OD ± SEM)	0.083 ± 0.05	0.028 ± 0.01	−0.945, 0.376
CAP-IgG4 (OD ± SEM)	0.019 ± 0.07	0.008 ± 0.02	−1.031, 0.355
SEA-IgG4 (OD ± SEM)	0.123 ± 0.05	0.019 ± 0.009	−1.714, 0.087
Derp-IgE (OD ± SEM)	0.049 ± 0.02	0.037 ± 0.007	−1.337, 0.190
Derp-IgG4 (OD ± SEM)	0.048 ± 0.016	0.243 ± 0.13	−1.286, 0.211
Schizont-IgM (OD ± SEM)	0.098 ± 0.01	0.145 ± 0.02	−1.901, 0.056
Schizont-IgG (OD ± SEM)	0.058 ± 0.02	0.028 ± 0.009	−0.968, 0.343
DD2-IgG (OD ± SEM)	0.034 ± 0.007	0.149 ± 0.09	−1.600, 0.119
MSP119-IgG (OD ± SEM)	0.029 ± 0.01	0.013 ± 0.004	−0.402, 0.721
CH150-IgG (OD ± SEM)	0.018 ± 0.005	0.005 ± 0.003	−0.987, 0.343

Comparison of treated and untreated groups in terms of baseline infection levels, demographic, and immunological characteristics. OD: optical density, SEM: standard error of mean. The *Z* and *P* values were calculated using the Mann-Whitney *U* (2 samples) test, and prevalences were compared using the *χ* ^2^ test. The significant value is highlighted in bold.

**Table 2:** Results from the non-parametric Wilcoxon signed-rank tests.

	Antibody isotype	Treated group	Untreated group
	CAP-IgM	***−4.037 (<0.000)***	−0.357 (0.36)
	SWAP-IgM	***−2.626 (0.0045)***	−0.533 (0.297)
	SEA-IgM	−0.987 (0.162)	−0.357 (0.361)
	CAP-IgE	−0.683 (0.247)	−0.296 (0.383)
Antischistosome responses	SWAP-IgE	***−2.633 (0.004)***	−0.968 (0.166)
	SEA-IgE	***−2.747 (0.003)***	−1.599 (0.055)
	CAP-IgG4	−0.35 (0.363)	−0.106 (0.458)
	SWAP-IgG4	**−2.018 (0.022) **	−0.415 (0.339)
	SEA-IgG4	−0.718 (0.237)	***−2.601 (0.003)***

Antiallergen responses	Derp-IgG4	***−3.746 (<0.000)***	**−1.96 (0.025)**
	Derp-IgE	***−3.497 (<0.000)***	−0.968 (0.166)

	Schizont-IgM	**−2.308 (0.01)**	**−1.992 (0.023)**
Antiplasmodium responses	Schizont-IgG	**−2.222 (0.013)**	−0.734 (0.231)
	CH150-IgG	**−2.103 (0.018)**	−1.473 (0.705)
	MSP1_19_-IgG	**−2.043 (0.021)**	**−1.897 (0.029)**
	Dd2-IgG	***−3.333 (0.0005)***	**−1.992 (0.023)**

*Z* and *P* values from the nonparametric Wilcoxon signed-rank tests comparing pre- and posttreatment antibody levels are shown for each antibody isotype. Significant results (*P* < 0.05) are highlighted in bold and those significant after Bonferroni-adjustmentin italic font.CAP: cercariae antigen preparation, SWAP: soluble worm antigen preparation, and SEA: soluble egg antigen.

**Table 3:** Potential identities of the proteins recognized.

Band	Protein name	Species	NCBI accession no.	Hit score	Mass
	Putative glycogen phosphorylase	*S. mansoni *	gi∣360045358	564	94169
	Surface protein	*S. mansoni *	gi∣501209	182	190732
~80 kDa	**Myosin heavy chain**	*S. mansoni *	gi∣256086969	159	171563
	**Actin**	*S. mansoni *	gi∣256084607	138	35652
	**Putative heat shock protein 70**	*S. mansoni *	gi∣353229993	125	70215
	Hypothetical protein Smp_208030	*S. mansoni *	gi∣353231600	53	333514

~100 kDa	GPI-anchored surface glycoprotein	*S. mansoni *	gi∣256052078	129	187956
	Putative glycogen phosphorylase	*S. mansoni *	gi∣360045358	100	94169

The 2 bands on silver stain that matched those identified on immunoblots were excised and subjected to trypsin digestion before being analysed by mass spectrometry. The list of Schistosome sequences (from NCBI database) that matched the peptide masses on the bands is indicated with the corresponding ions scores. Ions score is 10 ∗ Log (*P*), where *P* is the probability that the observed match is a random event. Individual ions scores >41 indicate identity or extensive homology (*P* < 0.05). The highlighted proteins are those previously recognized by pooled sera from adults.
